# Synthesis of novel thermotropic liquid crystalline polymers by a reactive extrusion process†

**DOI:** 10.1039/c8ra10410g

**Published:** 2019-04-17

**Authors:** Kyunghwan Oh, Hoyeon Kim, Yongsok Seo

**Affiliations:** RIAM, School of Materials Science and Engineering, College of Engineering, Seoul National University Kwanakro-1, Kwanakgu Seoul 151-744 Republic of Korea ysseo@snu.ac.kr

## Abstract

The syntheses of new thermotropic liquid crystalline polymers (TLCPs) were carried out *via* the polyaddition reactions of diepoxy-containing mesogens and a monoamine (1-naphthylamine). Both bulk polymerization and reactive extrusion were tested. The reaction between the two epoxy rings on the mesogen unit and the primary amine produces a thermotropic liquid crystalline polymer in the extruder. The amine group combines with the two epoxy rings in a single step *via* a polyaddition reaction to produce thermotropic liquid crystalline polymers without the formation of any by-products. Both polymers were found to exhibit nematic mesophase characteristics, which were examined by using polarized optical microscopy. The new thermotropic liquid crystalline polymers obtained with the bulk reaction have high molecular weights, whereas the polymers synthesized by using reactive extrusion have low molar mass due to their short residence times in the extruder. All the synthesized TLCPs were found to exhibit high thermal stability. Their decomposition temperatures were found to be above 350 °C, but their melting temperatures are low (below 250 °C). The liquid crystalline structures of the TLCPs were verified by performing 2D X-ray diffraction measurements. Scanning electron micrographs of the drawn polymer fibers show that the orientation of their morphologies lies predominantly along the direction of the fibers. The polymers synthesized with the reactive extrusion process have the same physical properties as those obtained with the bulk polyaddition reaction. This observation demonstrates the feasibility of the mass production of new TLCPs through reactive extrusion.

## Abstract 2

The syntheses of new thermotropic liquid crystalline polymers (TLCPs) were carried out *via* the polyaddition reactions of diepoxy-containing mesogens and a monoamine (1-naphthylamine).

## Graphical abstract

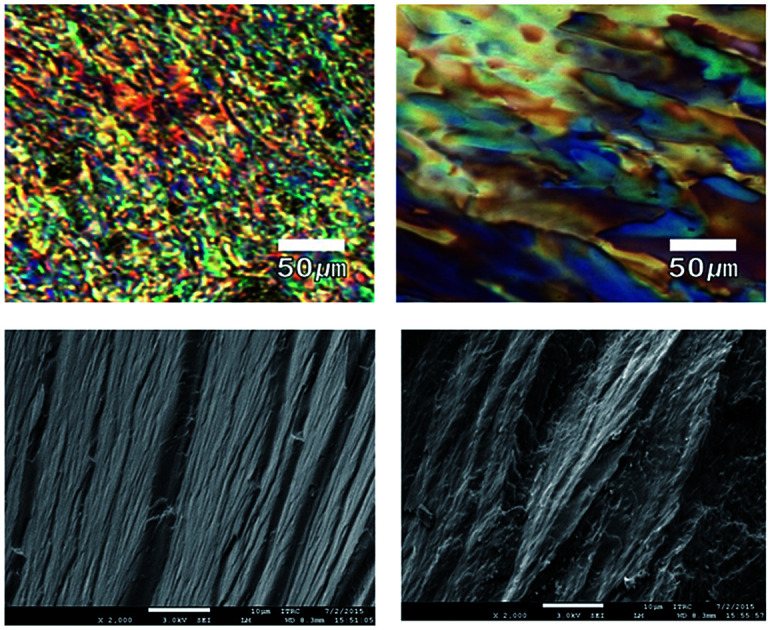

## Introduction

The outstanding physical properties of thermotropic liquid crystalline polymers (TLCPs) such as their high rigidity, high strength, excellent dimensional stability, high heat and chemical resistance, and exceptional barrier properties mean that they are super-engineering materials with a wide range of applications.^1–5^ These excellent properties are due to their unique structures, which arise from their mesogenic moieties that retain liquid crystalline order in the melt state. As a result, thermotropic liquid crystalline polymers (TLCPs) form a liquid crystalline structure in the melt state. Thus, they can be used in the production of so-called *in situ* composites with common engineering thermoplastics by using conventional processing equipments.^6–16^ However, most TLCPs have high thermal stability and high melting temperature, which means their processibility is not easy. Some TLCPs degrade before melting because their melting temperatures exceed their decomposition temperatures. The high processing temperatures of such TLCPs means that it is difficult to compound them with other common thermoplastic polymers to prepare *in situ* composites; such composites require thermoplastic matrices that can withstand the high transition temperatures or processing temperatures needed to convert the TLCP phase into fibril shapes for thermoplastic matrix reinforcement.^6–18^ Most TLCPs are synthesized *via* condensation polymerizations, which generally result in low molecular weights. Increasing the molecular weight of a TLCP improves, its mechanical properties, but also increases its melting temperature. Therefore, TLCPs with high molecular weights but low melting or processing temperatures are required. TLCPs with low transition or processing temperatures have been sought for the past 20 years.^19–22^ We have previously reported a new and simple process for the preparation of a novel type of thermotropic liquid crystalline polymers *via* a polyaddition reaction.^19^ This process consists of the reaction between a diepoxide compound with a primary monoamine (aniline) to produce a linear chain connecting the two epoxy groups. This approach can produce high molar mass TLCPs that have a low processing temperature because no by-products are generated that must be removed. By varying the structure of the diepoxy compound, the processing temperature of the resulting TLCP can be controlled. In this study, new TLCPs were synthesized by performing both reactive extrusion processes and bulk polymerizations in a flask. To the best of our knowledge, this is the first report of the synthesis of new TLCPs *via* a reactive extrusion process, which enables TLCP mass production in a single step. Mass production is feasible because these TLCPs are synthesized *via* a polyaddition process that does not generate any by-products that require removal. The processing temperature of the TLCPs can be optimized by varying the structure of the diepoxy compound. Hence, our approach enables the achievement both goals simultaneously.

## Experimental

**Scheme 1:**
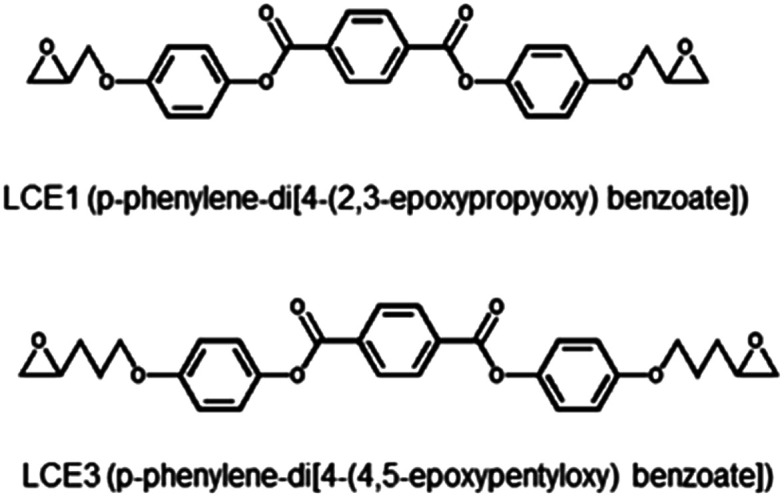
Chemical structures of LCE1 and LCE3.

Two diepoxides, LCE1 (*p*-phenylene-di-4-(2,3-epoxypropyloxy) benzoate) and LCE3 (*p*-phenylene-di-4-(4,5-epoxypentyloxy) benzoate) (Scheme 1), were prepared by using a reported procedure.^22^ All chemicals were purchased from Aldrich-Sigma Korea. The synthesis procedure can be simply recapitulated as follows: A mixture of 3-bromopropene, ethyl-4-hydroxybenzoate, K_2_CO_3_, and acetone was refluxed for 24 h. The solids were filtered off and the solvent was evaporated, then diethylether and water were added to the remnant. The diethylether layer was separated and washed three times with a 10% NaOH solution. After evaporation of the diethylether, the residue was boiled in an ethanol/water (1 : 2) solution containing KOH until the solution became clear. The pH was lowered to 2 by adding 2 N HCl solution. After filtration and washing with excess water, the solids were recrystallized in ethanol to yield 4-(2-propyloxy) benzoic acid as a white powder. A mixture of 4-(2-propyloxy) benzoic acid and SOCl_2_ was refluxed for 2 h. After evaporation of excess SOCl_2_, pyridine and hydroquinone were added. The mixture was reacted in an acidified aqueous solution, then filtered and washed with a 5% Na_2_CO_3_ aqueous solution and water. After drying, the solid product (LCE1) was recrystallized in ethyl acetate. LCE1 was purified with column chromatography by using CH_2_Cl_2_ as eluent. After evaporating the solvent, the LCE1 product was recrystallized in ethyl acetate with a yield of 55%. LCE3 was synthesized by using 5-bromopentene instead of 3-bromopropene. The yield of LCE3 was 69%. More details of the synthetic procedure are provided in the ESI.†

**Scheme 2:**
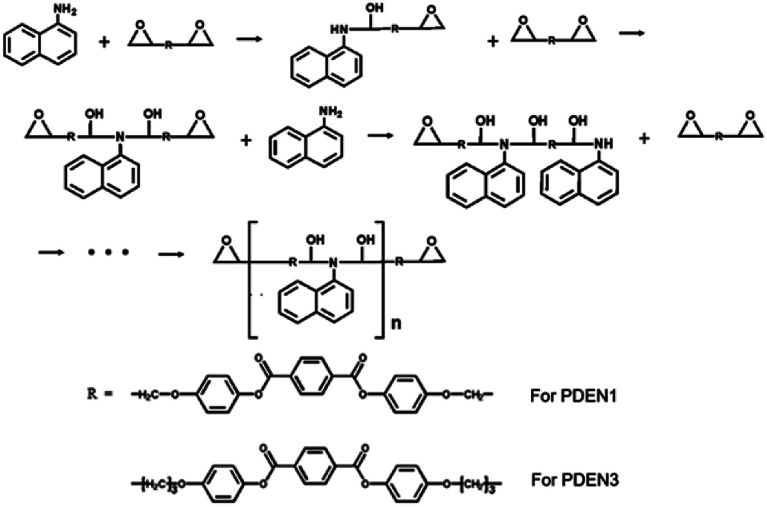
Synthesis of PDEN1 and PDEN3 *via* a polyaddition reaction.

Poly(diepoxy naphthalene)s (PDENs, Scheme 2) were first prepared in a flask by performing the melt polymerizations of the synthesized monomers (LCE1 and LCE3 have propyl and pentyl epoxy groups respectively) with a primary monoamine (1-naphthylamine). The reactive extrusions were carried out at a fixed rotation speed of 30 rpm in a 42 mm Brabender twin screw extruder (AEV651). The extrusion temperatures of the feeding zone/transporting zone/melting zone/die were set as 140/220/220/210 °C, respectively.

Films of the resulting polymers (PDEN1, PDEN1R (PDEN1 synthesized with the reactive extrusion process), PDEN3, and PDEN3R (PDEN3 synthesized with the reactive extrusion process)) were characterized by performing Fourier transform infrared spectrometry (FT-IR, Nicolet 6700, Thermo Scientific (USA)) with an attenuated total reflection (ATR) accessory. The spectra were compiled from 32 scans at a resolution of 8 cm^−1^. Prior to these measurements, the TLCP samples were hot pressed at 250 °C and cooled to room temperature to obtain the polymer films.

^1^H-NMR and ^13^C-NMR spectra were obtained with a Bruker spectrometer (300 MHz). The samples were prepared by dissolving the polymers in the solvent THF-d_8_. The ^1^H-NMR spectra were referenced to residual TMS (0 ppm) except in the case of D_2_O (solvent reference, 4.79 ppm) and DMF-d_7_ (solvent reference, 8.03 ppm). The chemical shifts of the ^13^C-NMR spectra were measured relative to DMF-d_7_ (67.21 and 25.31 ppm).

The thermal properties of the TLCPs, namely their degradation temperatures and transition behaviors, were analyzed by using thermogravimetric analysis (TGA, TGA/DSC1, Mettler Toledo) and differential scanning calorimetry (DSC, DSC 823e, Mettler Toledo). Under a nitrogen atmosphere, the samples were heated in the TGA measurements from 25 to 450 °C at 10 °C min^−1^. In the DSC measurements, each sample was first heated from 25 to 320 °C at 10 °C min^−1^, cooled to 25 °C, and then heated to 320 °C again at the same heating rate under a nitrogen atmosphere.

Gel permeation chromatography (GPC, Viscotek model 250) was performed with an RI750F refractive index detector. Waters Styragel HP 4E and Styragel HR 5E columns were used with THF as the eluent at a flow rate of 1 mL min^−1^ at 40 °C. Polystyrene standards were used for column calibration.

Polarized light optical microscopy (POM, BX51, Olympus) was used in the polarization mode to observe the thermal transition behaviors of the polymers. The specimens were prepared by hot pressing the samples between two slide glasses at 250 °C. The measurement temperature was controlled in the range 25 to 320 °C with a Mettler Toledo FP82HT hot-stage. The polymers were extruded by using a mini-extruder (DACA 5000) with a pulling unit. Each specimen's morphology was examined with scanning electron microscopy (SEM, Hitachi S 2200C). The surface of each sample fractured in liquid nitrogen was coated with Pt/Pd before the SEM examination.

2-D high temperature X-ray diffraction (XRD) measurements were carried out with a D8 Discover (Bruker) by using the General Area Detector Diffraction System (GADDS) program. XRD scans were performed with CuKα radiation from 25 to 230 °C.

## Results and discussion

**Fig. 1:**
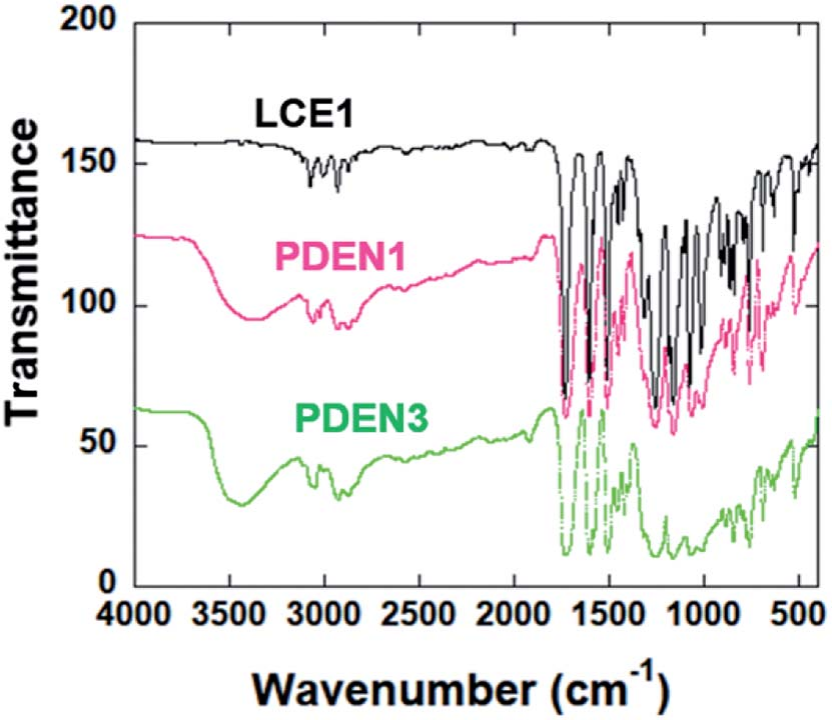
FTIR of the LCE1, PDEN1 and PDEN3

Fig. 1 shows the FT-IR spectra of the polymers. The sharp absorption peak at 914 cm^−1^ is due to the vibration of the oxirane ring in LCE1. The reaction between the amine group of 1-naphthyl amine and the oxirane ring (Scheme 2) results in the appearance in the TLCP spectra of a broad shoulder absorption peak at 3450 cm^−1^, which is ascribed to the stretching vibrations of the produced OH groups. The intensity of the peak due to the oxirane ring in the TLCPs is significantly reduced.^16^ The epoxide groups on LCE1 and LCE3 react first with the amine group of 1-naphthylamine and then next with the resulting secondary amine to form linear chains (PDEN1 and PDEN3 in Scheme 2). These steps can be repeated alternately to generate high molecular weight polymers.

**Fig. 2:**
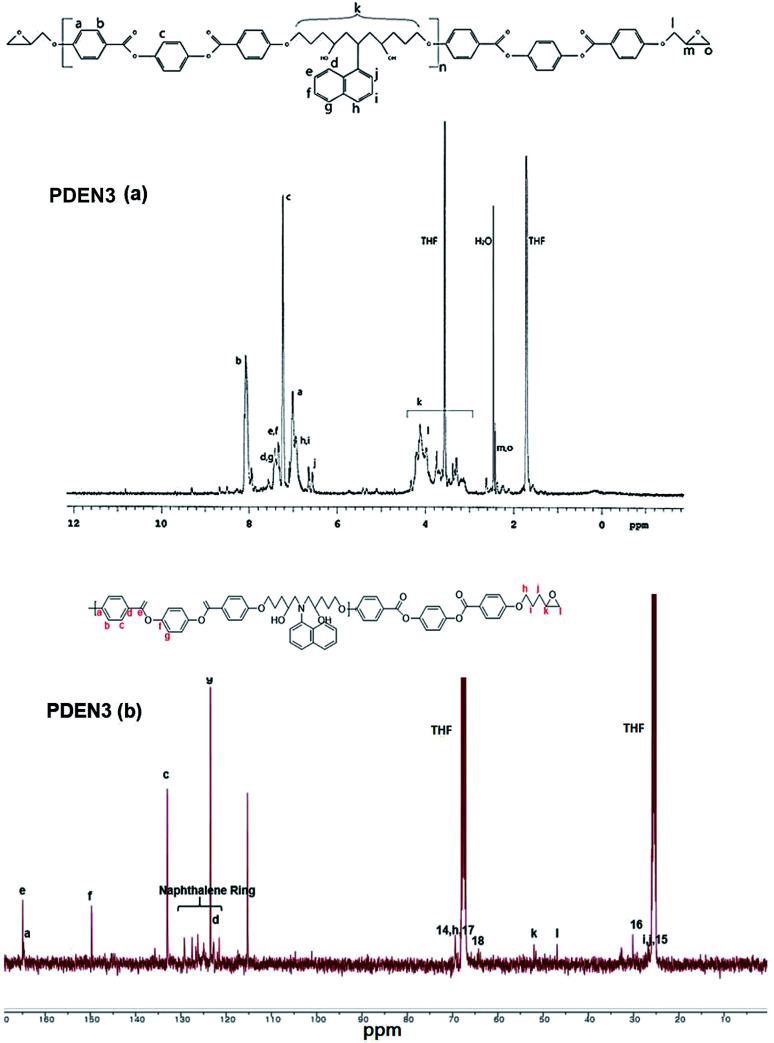
(a) ^1^H NMR of PDEN3, (b) ^13^C NMR of PDEN3.

The ^1^H NMR spectra of the polymers (PDEN1, PDEN3, PDEN1R, and PDEN3R) were analyzed by using Chemsketch software. The assignment of the signals to the corresponding protons was performed to determine the polymer structures. The mesogenic monomer LCE1 contains five protons on its phenyl rings and three protons on its two aliphatic groups, including those on the methyl groups of the spacer unit and the oxirane ring. All the protons were uniquely identified (Fig. 2(a)). The spectra of PDEN1 and PDEN1R are identical, which confirms that they have the same structure (ESI Fig. 3a and b).† The aromatic proton signals arise at 6.5–8.2 ppm, and the methyl group signals of the TLCPs appear at 3.2–5.3 ppm. The corresponding protons of the LCEs appear at 2.5 and 4.1 ppm respectively.^16^ The reaction between the primary amine group and the epoxy ring proceeds through a ring-opening reaction *via* nucleophilic attack on the amine, which is accompanied by the appearance of new peaks at 4.7 and 5.3 ppm. The doublet at 3.5 ppm is due to the hydrogen atoms in 1-naphthylamine and disappears as the reaction proceeds.

The positions of the peaks in the ^13^C NMR spectrum of PDEN1 are listed in ESI Fig. 3 and Table 1.† These characteristic peaks are consistent with those of the polymer structures. The characteristic aromatic naphthalene peaks appear at 150.1 and 150.5 ppm, which confirms the formation of PDEN1. These spectral results clearly confirm the polymer structures in Scheme 2. As mentioned above, the first epoxy group reacts vigorously with the amine group to form a compound containing a secondary amine that further reacts with another epoxy group to form a linear polymer chains (Scheme 2). Although the reactivity of the secondary amine group is known to be lower than that of the primary amine groups, it reacts rapidly to form the polymers.^21,22^

The number and weight average molar masses obtained by using gel permeation chromatography are listed in Table 1. The weight average molar masses of the bulk synthesized polymers (PDEN1 and PDEN3) are relatively high, although the polydispersity index (PDI) values of PDEN1 and PDEN3 are 2.0 and 3.2, respectively. PDEN3 has a lower molar mass than PDEN1 because the diffusivity of the LCE3 monomer is lower than that of the LCE1 monomer, as found in our previous study.^19^ These large PDI values indicate that the molar mass distribution of the TLCPs are quite broad. In bulk polymerizations, the viscosity increases as the polymerization progresses, which interferes with the diffusion of the LCEs. The polymers synthesized with the reactive extrusion process exhibit better mixing, but the short residence time in the extruder does not allow sufficient reaction time for the preparation of high molar mass polymers. However, the reactive extrusion of LCE1 does result in polymers with a higher molar mass than those of LCE3. The low boiling point of aniline (184 °C) means that it cannot be used in reactive extrusion processes whereas 1-naphthylamine (b.p. 301 °C) can be. PDEN1 and PDEN3 have higher molar masses than the other TLCPs since they were prepared *via* polyaddition reactions without any by-products.

**Table 1:** Molar mass and the thermal properties of the polymers^a^

Samples	*M*_n_	*M*_w_	PDI	*T*_g_ (°C)	*T*_m_ (°C)	*T*_dg_ (5 wt% °C)
PDEN1	16 200	32 400	2	100	175	357
PDEN1R	10 400	18 200	1.8	95	182	352
PDEN3	14 900	46 500	3.2	71	193	358
PDEN3R	8600	13 600	1.6	73	194	358

a *T*_g_: glass transition temperature, *T*_m_: melt temperature, *T*_dg_: 5 wt% degradation temperature.

**Fig. 3:**
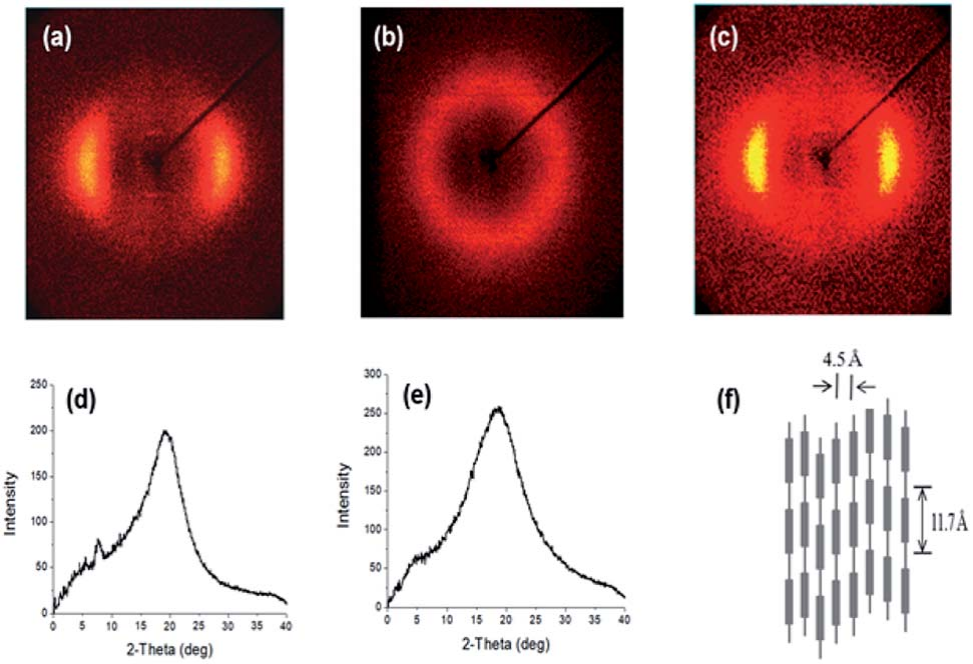
XRD patterns of (a) PDEN1 at 170 °C, (b) PDEN1 at 230 °C, (c) PDEN1R at 170 °C, (d) PDEN1 at 170 °C, (e) PDEN1 at 230 °C, (f) schematic of PDEN1 LC ordering.

The crystalline structures of the synthesized polymers were investigated with 2-D wide angle X-ray diffraction; the resulting patterns are shown in Fig. 3 and contain inner layer reflections on the meridional scan at a temperature below *T*_m_. As for PDEA1, which was synthesized *via* the reaction between LCE1 and aniline in our previous study, a sharp inner peak is evident at a diffraction angle of 2*θ* = 7°, which corresponds to a packing distance of 11.7 Å according to Bragg's law. Wider outer peaks can also be seen at a diffraction angle of 2*θ* = 19–20°, which corresponds to equatorial scans of 4.5–4.7 Å. Since we used the same mesogenic units (LCE1 and LCE3) as before, their diffraction patterns are quite very similar.^19^ LC chains typically produce diffraction peaks corresponding to a lateral-packing of 3–6 Å.^1,23,24^ The peaks in the wide angle region are broader than those of PDEA1 and PDEA3, which indicates that the ordering of the PDEN1 and PDEN3 chains is more liquid-like (less ordered) due to their bulkier side groups. As the temperature rises to 230 °C, the small angle reflections disappear while the broad peak at wide angles remains intact, which means that there is a breakdown in chain regularity and in the nematic phase output at that temperature.^25^

**Fig. 4:**
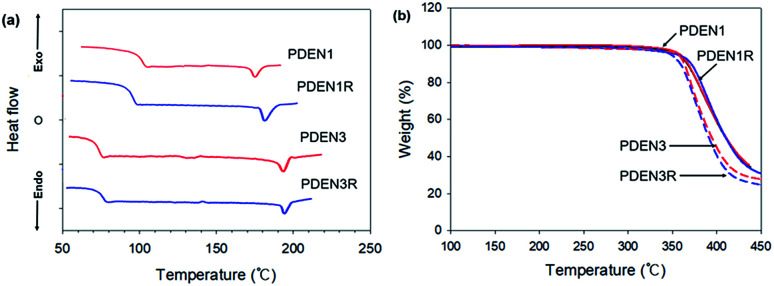
(a) DSC thermograms and (b) TGA degradation curves.

The thermal behaviors of the polymers were investigated by using the DSC and TGA. A single endothermic melting point appears in each of the DSC thermograms during the heating scans (Fig. 4(a)). By recording second heating thermograms to remove the complex thermal histories of the samples, we were able to determine their glass transition temperatures (*T*_g_), and melting temperatures (*T*_m_), which are listed in Table 1. Above *T*_g,_ chain relaxation and flexibility become evident. Increasing the length of the flexible spacer in the main chain reduces the rigidity of the polymer backbone by decreasing the proportion of the mesogen unit.^19^ Thus PDEN3 has a lower *T*_g_ than PDEN1 because of its soft backbone, but has a higher *T*_m_ due to the greater ordering of its alkyl groups. Both polymers, however, have a low melting temperature (175 °C for PDEN1 and 194 °C for PDEN3), so they can easily be processed in an extruder or injection molding machine. Table 1 also provides the degradation temperatures (*T*_dg_ of 5 wt% loss) of the polymers. The TGA thermograms in Fig. 4(b) show that all polymers have high thermal stabilities. Their degradations starts at temperatures above 350 °C and proceeds in a single stage, which indicates that the presence of the flexible spacer groups does not affect the high thermal stability of the lengthy cores with ester linkers.

**Fig. 5:**
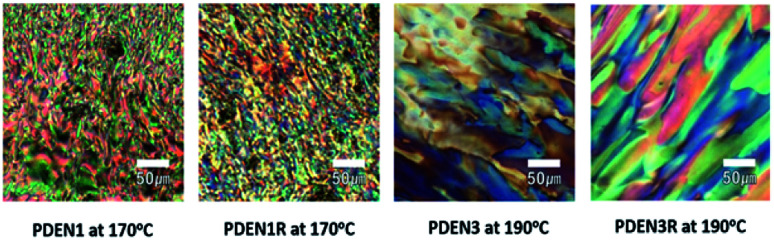
POM images of PDEN1, PDEN1R, PDEN3, and PDEN3R respectively.

**Fig. 6:**
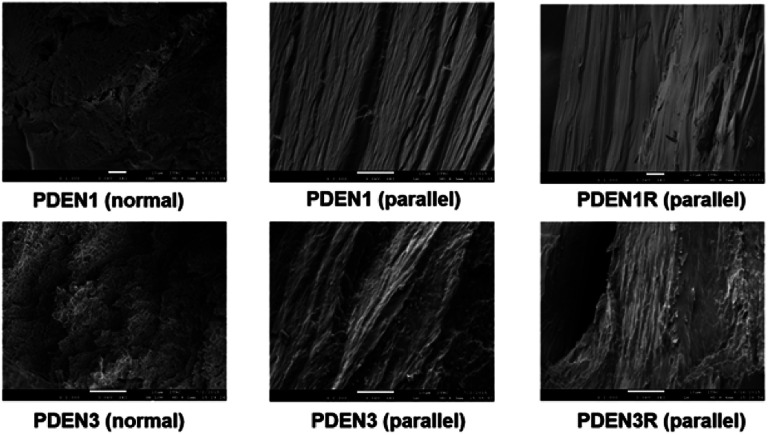
SEM micrographs of fractured polymer fibrils (upper row: PDEN1 fractured surface normal to fibril direction (left); parallel to fibril direction (center); PDEN1R fractured surface parallel to fibril direction (right); lower row: PDEN3 fractured surface normal to fibril direction (left); parallel to fibril direction (center); PDEN3R fractured surface parallel to fibril direction (right)) (×2000 magnification).

The optical textures of the polymers near the transition temperature are presented in Fig. 5. Both polymers exhibit the birefringence characteristics of nematic liquid crystalline mesophase textures (the marbled nematic mesophase) near the transition temperature.^26^ Since these images were collected during the second heating process, they have characteristic birefringent textures even at temperatures lower than the transition temperature. The colorful textures remain almost unchanged when the samples are cooled to 25 °C after the first heating, which means that the mesophases of the polymers formed at high temperatures are stable. Fibril formation was confirmed with SEM, by examining the surface morphologies of the melt-drawn fibers (Fig. 6). All the samples contain well-oriented TLCP fibrils. Protruding microfibrils can be seen in the cross-sections of the fibers after cryogenic fracturing in liquid nitrogen. These fibrils protrude above the fractured surfaces, and are thus part of continuous fibril morphologies on the TLCP surfaces that are oriented parallel to the fibril direction.^13,19,27^

## Conclusions

New liquid crystalline polymers with low melting temperatures have been successfully synthesized by performing polyaddition reactions between diepoxide molecules and a primary monoamine (1-naphthylamine). Although the polymers synthesized *via* the reactive extrusion process have lower molar masses than the same polymers prepared in bulk reactions due to their short reaction time in the extruder, this approach is commercially very significant because of its potential for the mass production. The polymers synthesized with bulk polymerizations have high molar masses, which means that it is possible to produce high molar mass TLCPs in a commercial extruder that provides sufficient residence time for the reactants.

The polymers have good thermal stabilities and high decomposition temperatures above 350 °C, which means that they can withstand high temperature applications. They also have low melting temperatures, which enables easy processing. PDEN3 has longer spacer groups and thus a high transition temperatures, but the thermal stabilities of the two polymers (PDEN1 and PDEN3) are comparable because the spacer groups are not so long (methyl and propyl). The polymers synthesized with the reactive polymerization process have nearly the same thermal properties as those synthesized with bulk polymerization. All the polymers exhibit a nematic liquid crystal mesophase morphology in the melt state. The polymers were found to form well-ordered structures upon cooling. After drawing, the stretched polymer fibers exhibit strong molecular orientation on the surface along the direction of flow. The many microfibrillar structures that protrude out perpendicular to the direction of flow from the fractured surface mean that these nematic polymers can be used in the preparation of *in situ* composites.

## Conflicts of interest

The authors declare that there are no conflicts of interest.

## Supplementary Material

RA-009-C8RA10410G-s001
