# Supplementary material for: Synthesis of novel thermotropic liquid crystalline polymers by a reactive extrusion process
Source: RSC Adv. 2019 Apr 17;9(22):12189–94. doi: 10.1039/c8ra10410g (PMC9063539; doi:10.1039/c8ra10410g)
Supplement: RA-009-C8RA10410G-s001 [file RA-009-C8RA10410G-s001.pdf]

Electronic Supplementary Material (ESI) for RSC Advances.
This journal is © The Royal Society of Chemistry 2019

Supplementary Information

SI 1. Synthesis of p-Phenylene-di[4-(2,3-epoxypropyloxy) benzoate] (LCE1)

Synthetic scheme was reported by Jun Yeob Lee.[Lee, J. Y.; Jang, J. S. *Polymer* 2006, 47, (9), 3036-3042.] A mixture of ethyl-4-hydroxybenzoate (0.6 mol, 99 g), 3-bromopropene (0.75mol, 90 g), K_2CO_3 (0.9 mol, 126 g), and 800 ml of acetone were refluxed for 24 h. After the filtering off the solid, the solvent was evaporated away. Diethylether 400 mL and water 200 mL were poured into the remaining solution. The diethylether layer was separated and washed with 200 mL of a 10% NaOH solution three times. After evaporating away the diethylether, the residue was boiled in 900 mL of an ethanol/water (1:2) solution containing 90 g KOH until the solution was cleared. A 2N HCl solution was added to reach a pH of 2. After filtration, the solids were washed with excess water and recrystallized in ethanol to provide (4-(2-propyloxy) benzoic acid) as a white powder. A mixture of 4-(2-propyloxy) benzoic acid (0.11 mol, 21 g) and $SOCl_2$ (0.4 mol, 29 mL) was refluxed for 2 h. After evaporating excess $SOCl_2$, 80 mL of pyridine were poured into the solution. Hydroquinone (0.55 mol, 6.3 g) was then added. The mixture was reacted in 1L of an acidified aqueous solution, filtered and washed with 500 mL of a 5% Na_2CO_3 aqueous solution and 500 mL of water. After drying, the solid product (LCE1) was recrystallized in ethyl acetate. LCE1 was purified by column chromatography using CH_2Cl_2 as an eluent solvent. After evaporating away the solvent, the LCE1 product was recrystallized in ethyl acetate. The yield was 55%. ^{1}H-NMR spectrum ($CDCl_3$): δ (ppm) 8.13 (4H, d), 7.23 (4H, s), 6.99 (4H, d), 4.33 (2H, dd, CH_2 of glycidyl), 4.02 (2H, dd, CH_2 of glycidyl), 3.38 (2H, s, CH of epoxy), 2.92 (2H, dd, CH of epoxy), 2.78 (2H, dd, CH of epoxy).

SI 2. Synthesis of p-Phenylene-di[4-(4,5-epoxypentyloxy) benzoate] (LCE3)

Synthetic scheme was as same as LCE1, expect using 5-bromopentene instead of 3-

bromopropene. Transition temperatures are following. Crystal to smectic : 110℃, smectic to nematic : 139℃, nematic to isotropic : 235℃. Yield: 69%. H-NMR spectrum ($CDCl_3$): δ (ppm) 8.12 (4H, d), 7.24 (4H, s), 6.96 (4H, d), 4.11 (4H, m, CH_2 of glycidyl), 3.01 (2H, m, CH of epoxy), 2.78 (2H, m, CH of epoxy), 2.51 (2H, m, CH of epoxy), 2.01 (4H, m, CH_2 of glycidyl), 1.85 (2H, m, CH_2 of glycidyl), 1.67 (2H, m, CH_2 of glycidyl).

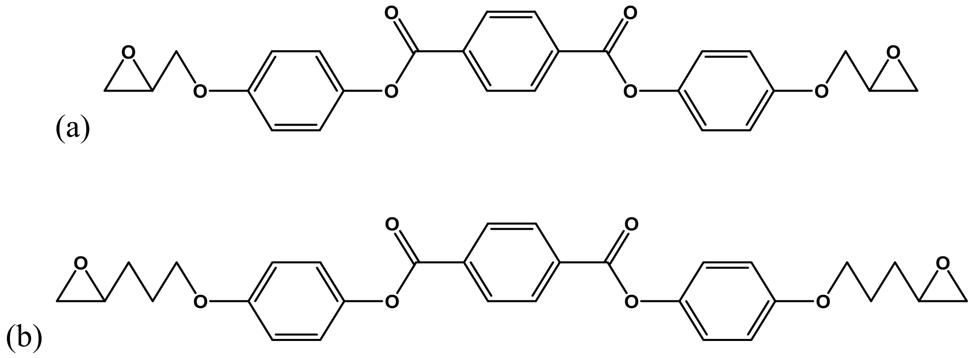

SI Figure 1 Chemical structure of LCE1 (a) and LCE3 (b).

SI 3. Polymerization and PDEN1 & PDEN3

Melt polymerization of the synthesized monomers (LCE1 and LCE3, having propyl epoxy and pentyl epoxy groups, respectively.) with a primary monoamine (1-naphthylamine) were first performed in a flask to produce poly(diepoxy naphthalene)s (PDENs, Scheme 2). The reactive extrusion was carried out in a 42 mm Brabender twin screw extruder (AEV651) at a fixed rotation speed of 30 rpm. The extrusion temperatures of the feeding zone/transporting zone/melting zone/die were set as 140/220/220/210 °C, respectively.

The resulting polymers (PDEN1, PDEN1R (PDEN1 synthesized by the reactive extrusion process), PDEN3, and PDEN3R (PDEN3 synthesized by the reactive extrusion process)) were characterized using Fourier transform infrared spectrometry (FT-IR, Nicolet 6700, Thermo Scientific (USA)) with ATR accessory. The spectra were recorded with 32 scans at 8 cm^{-1} resolution. Prior to measurement, TLCP samples were hot pressed at 250℃ and cooled to room temperature to obtain films.

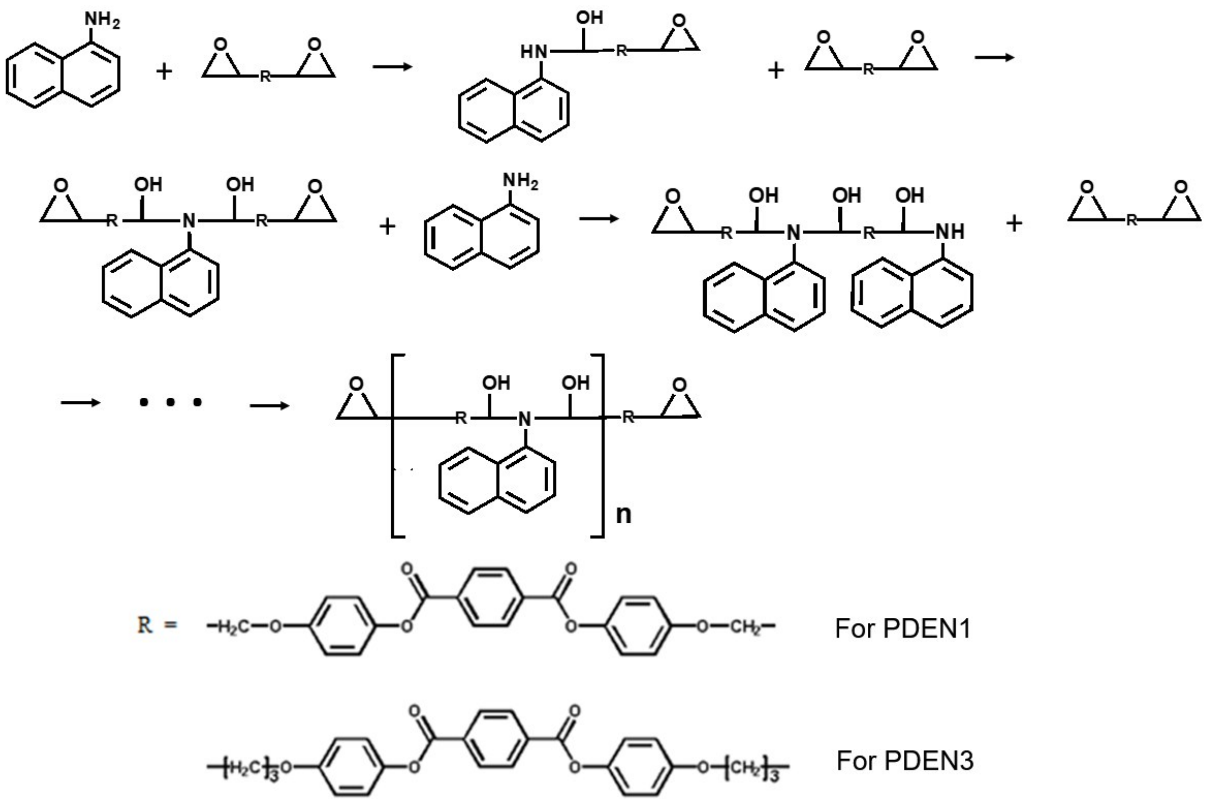

SI Figure 2 Synthesis of PDEN1 and PDEN3 via a polyaddition reaction

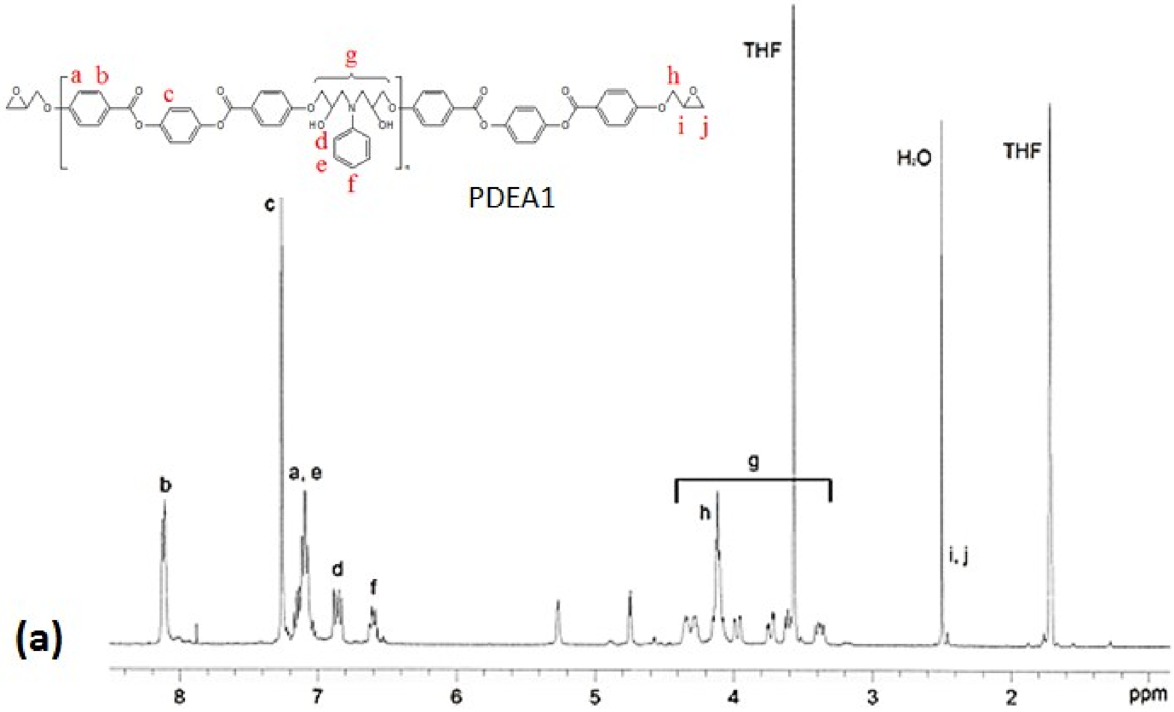
a b c g h i j d e f
PDEA1
THF
H2O
THF
c
b
a, e
d
f
g
h
i, j
(a)
8 7 6 5 4 3 2 ppm

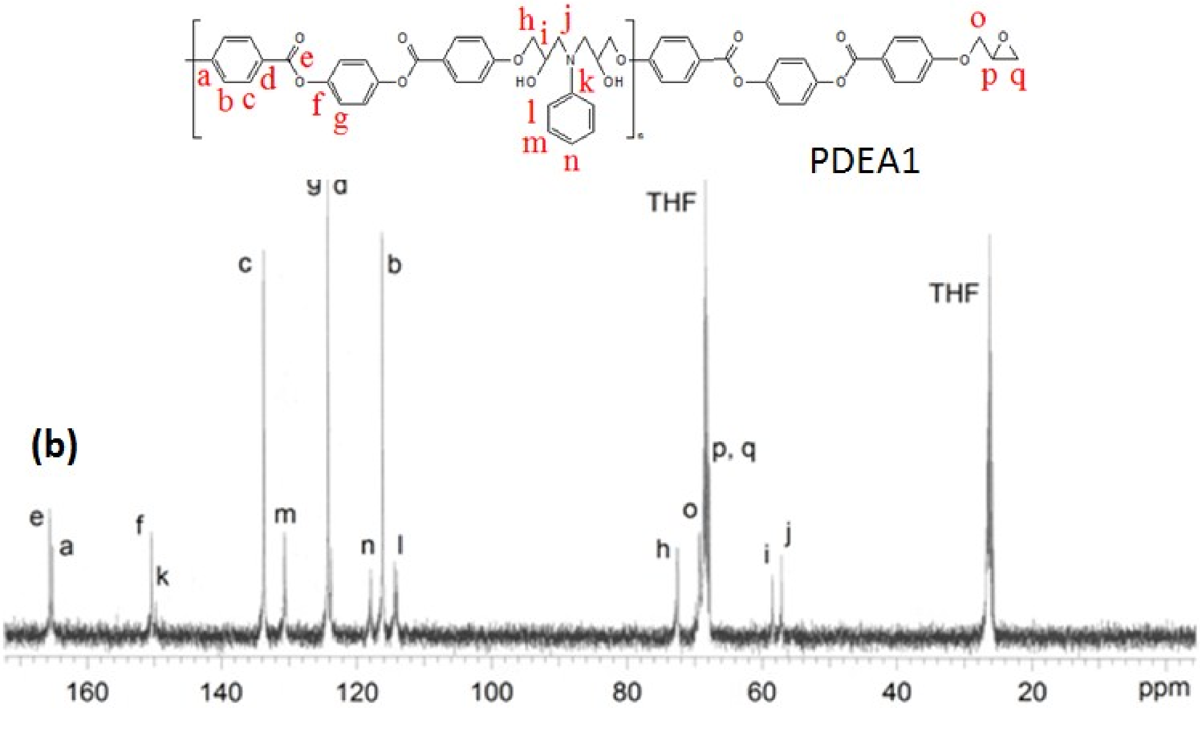
a b c d e f g h i j k l m n o p q
PDEA1
THF
THF
c
b
m
e
a
f
k
n
l
h
o
p, q
i
j
(b)
160 140 120 100 80 60 40 20 ppm

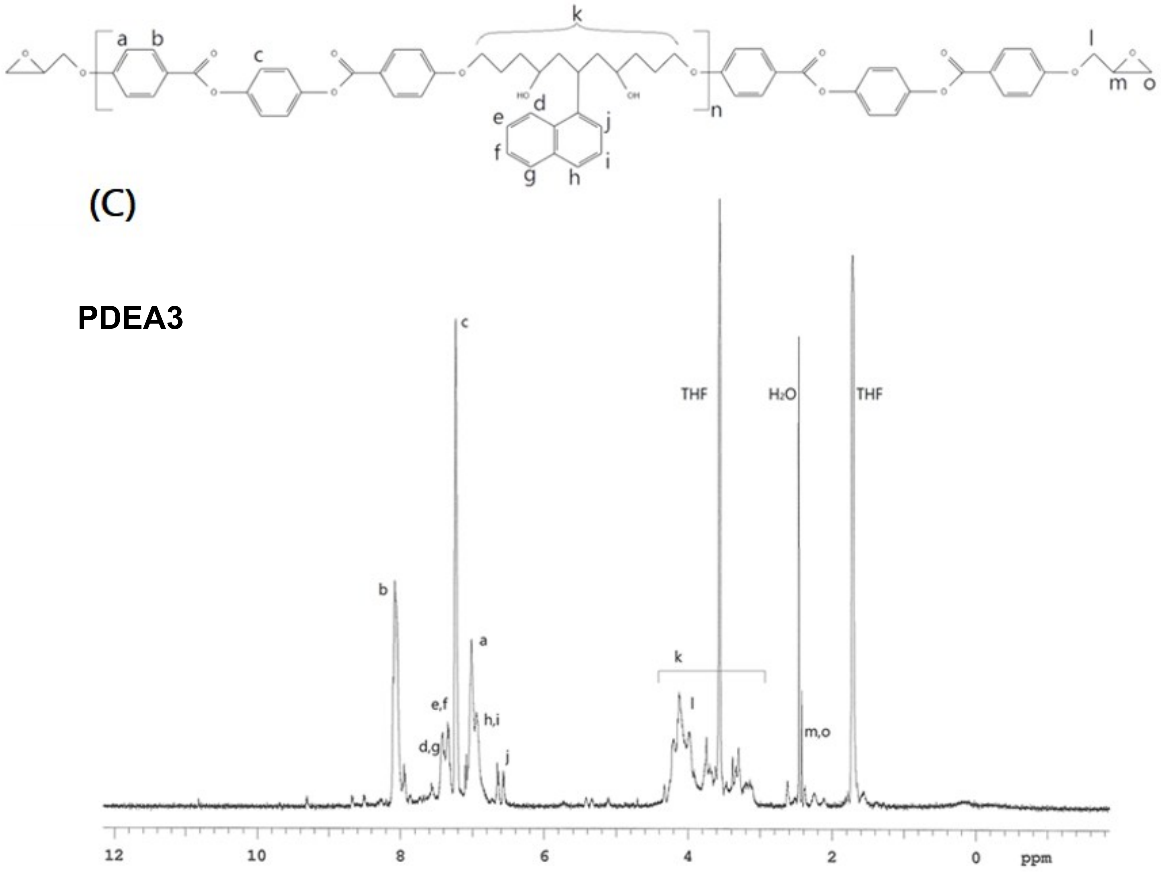

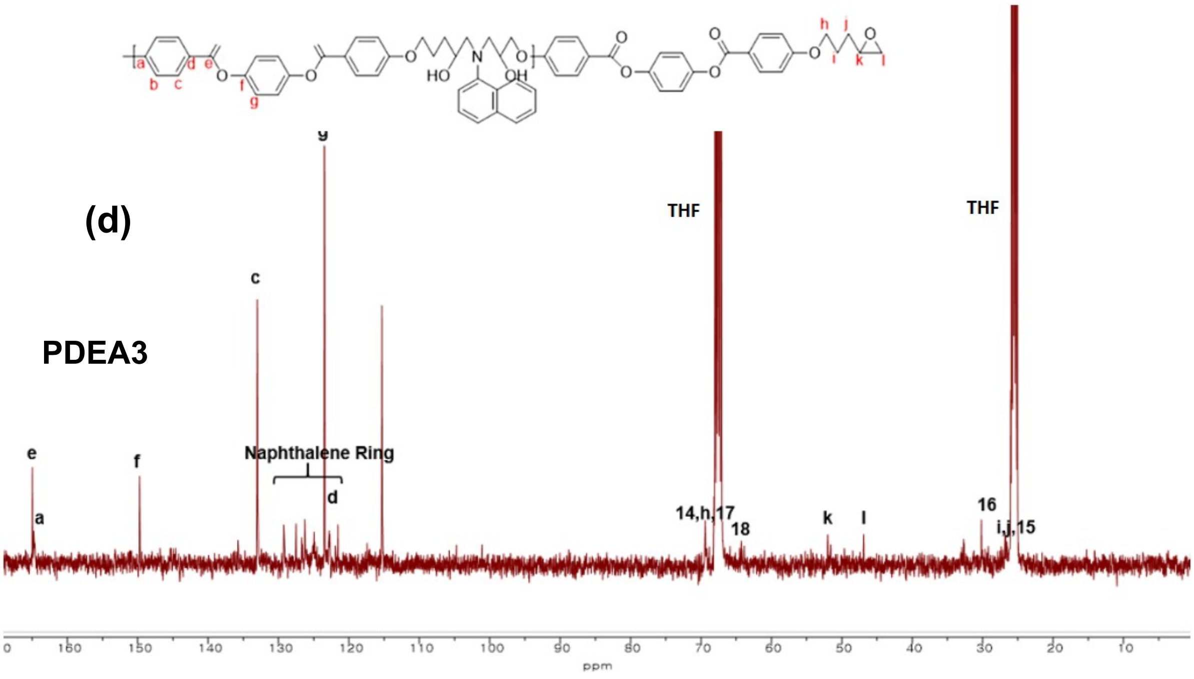

SI Figure 3. (a) ^{1}H NMR and (b) ^{13}C NMR of PDEA1 (c) ^{1}H NMR and (d) ^{13}C NMR of PDEA3

S. I. Table 1. ^{13}C-NMR spectra of LCPs (δ in ppm)

LCPs	**PDEN1, PDEN1R**	**PDEA3**	**PDEN3, PDEN3R**
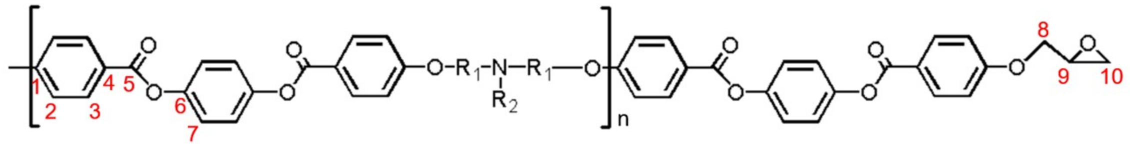			
**R_1**	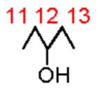	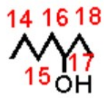	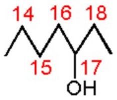
C atom **R_2**	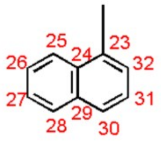	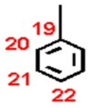	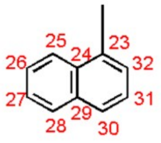
1	164.7	163.5	164.8
2	116.1	114.3	116.1
3	132.7	131.9	133.0
4	123.7	121.7	123.3
5	165.3	164.5	165.8
6	150.1	148.4	148.6
7	123.8	122.4	123.5
8	70.5	68.9	71.1
9	51.2	51.0	52.2
10	45.3	45.8	45.9
11	72.8		
12	68.3		
13	60.3		
14		68.6	69.5
15		24.5	25.8
16		31.2	31.7
17		68.0	68.8
18		66.2	65.0
19		149.2	
20		113.1	
21		128.9	
22		117.0	
23	150.1		150.5
24	132.4		132.8
25	125.7		125.0
26	127.3		127.1
27	127.5		130.0
28	129.3		131.0
29	136.4		136.5
30	124.3		124.3
31	128.1		130.3
32	117.2		117.2
